# MiR‐132 down‐regulates high glucose‐induced β‐dystroglycan degradation through Matrix Metalloproteinases‐9 up‐regulation in primary neurons

**DOI:** 10.1111/jcmm.16669

**Published:** 2021-06-23

**Authors:** Yunxiao Dou, Yan Tan, Tongya Yu, Xiaoye Ma, Yuchen Zhou, Yichen Zhao, Yanxin Zhao, Xueyuan Liu

**Affiliations:** ^1^ Department of Neurology Shanghai Tenth People's Hospital Tongji University School of Medicine Shanghai China

**Keywords:** cognitive dysfunction, dystroglycan protein, high glucose, Matrix metalloproteinases‐9, microRNA‐132, primary neurons

## Abstract

Cognitive dysfunction is one of the complications of diabetes. Unfortunately, there is no effective methods to block its progression currently. One of the pathophysiological mechanisms is synaptic protein damage and neuronal signal disruption because of glucose metabolism disorder. Dystroglycan protein, located in the post‐synaptic membrane of neurons, links the intracellular cytoskeleton with extracellular matrix. Abnormal expression of dystroglycan protein affects neuronal biological functions and leads to cognitive impairment. However, there are no relevant studies to observe the changes of β‐dystroglycan protein in diabetes rat brain and in primary neurons under high glucose exposure. Our data demonstrated the alterations of cognitive abilities in the diabetic rats; β‐dystroglycan protein degradation occurred in hippocampal and cortical tissues in diabetic rat brain. We further explored the mechanisms underlying of this phenomenon. When neurons are exposed to high glucose environment in long‐term period, microRNA‐132 (miR‐132) would be down‐regulated in neurons. Matrix Metalloproteinases‐9 (MMP‐9) mRNA, as a target of miR‐132, could be up‐regulated; higher expression and overlay activity of MMP‐9 protein could increase β‐DG protein degradation. In this way, β‐DG degradation may affect structure and functions among the synapses, which related to cognition decline. It may provide some theoretical basis for elucidating the molecular mechanism of diabetes‐induced cognitive dysfunction.

## 1 INTRODUCTION

Diabetes mellitus (DM) is concerned with cognitive dysfunction. Most studies reported the aggregate outcome that the incidence of ‘any dementia’ in diabetic patients is higher than that in non‐diabetic patients; many studies determined the intensity of the association between diabetes and dementia.^1^ Cognitive changes in patients with type 2 diabetes mainly include impaired memory and learning ability.^2^ Diabetes can affect the occurrence and development of dementia through a variety of pathophysiological mechanisms. Although the mechanisms are complex and diverse, it generally includes glucose toxicity, lipid toxicity, insulin resistance and so on.^2^, ^3^, ^4^ Early studies have reported that high glucose toxicity can slowly cause progressive structural and functional abnormalities in the brain.^5^ Several lines of evidence also suggested rodents suffered from chronic hyperglycaemia had abnormal synaptic plasticity and cognitive impairments.^6^ Thus, we concluded that toxic effects of high glucose on synaptic proteins may aggravate cognitive impairments. However, there is still many room for the high glucose effects on synaptic proteins.

There exists a large quantity of research on the role of dystroglycan in the function of neuromuscular synapses, opposed to a relatively small number of studies that deal with dystroglycan at inter‐neuronal synapses. Dystroglycan protein widely exists in cortex and hippocampus neurons in brain.^7^ Dystroglycan protein is located in the post‐synaptic membrane of neurons; the protein connects the intracellular cytoskeleton and extracellular matrix (ECM).^8^ The dystroglycan propeptide is translated from a single mRNA, which is subsequently cleaved into two non‐covalently associated subunits: extracellular‐dystroglycan (α‐DG) and transmembrane‐dystroglycan (β‐DG).^9^ The α‐DG is a kind of peripheral membrane protein, which can be connected to many extracellular matrix parts ^10^ and pre‐synaptic neurexins.^11^ The β‐DG connects dystrophin glycoprotein complex (DGC) to F‐actin cytoskeleton through proline‐rich C terminus.^12^, ^13^ Dystroglycan may play a key role in synaptic plasticity and cognition. It has been proved that mutant rats that are deficient in dystroglycan protein selectively have cognitive impairments and insufficient long‐term potentiation effect.^14^, ^15^ Dystroglycan protein may also be affected by hyperglycaemia. Recent studies reported that the β‐DG protein expression significantly reduced in skeletal muscle of Goto Kakizaki rat model of type 2 diabetes.^16^ However, there are no relevant studies to observe the changes of dystroglycan protein in neurons under the high glucose toxic.

MicroRNAs (miRNAs) are short non‐coding RNAs, which are important regulators for controlling many biological processes and post‐transcriptional gene expression. They usually bind specifically to the 3′ untranslated region (3′ UTR) of messenger RNA (mRNA) and collect RNA induced silence complex.^17^, ^18^, ^19^, ^20^ It has been found that miRNAs are particularly rich in brain and give scope to neuronal plasticity, including synaptic protein synthesis and senior functions of brain.^21^, ^22^, ^23^, ^24^

The miR‐132 is abundant in human and rat brain, especially in the hippocampi or cortices. Abnormal miR‐132 expression in a certain kind of cell or tissue may be involved in diseases processes.^25^ However, the expression of miR‐132 in neurons under the high glucose exposure has not been reported. Up to now, several targets of miR‐132 have been mentioned, including P300, MeCP2, Foxp2 and Matrix Metalloproteinases‐9 (MMP‐9).^26^, ^27^, ^28^, ^29^ The miR‐132 could pair to the 3' UTR of target mRNA thus promote mRNA degradation and repress translation.

Matrix Metalloproteinases (MMPs) are a family of proteolytic enzymes that are able to affect cell behaviour through cleaved structural components of various extracellular matrix molecules.^30^ The MMPs subfamily of gelatinases, especially MMP‐9 and Matrix Metalloproteinases‐2 (MMP‐2), is currently the widely studied. The expression level of MMP‐9 in hippocampus of adult rodent is especially high and MMP‐9 co‐localizes with pre‐synaptic and post‐synaptic markers.^31^ It is worth mentioning that dystroglycan and MMP‐9 proteins jointly play an essential role in neuronal function. On the one hand, the gene absence of dystroglycan gene (DAG1) in the brain leads to neuronal plasticity defect, similar to the inhibition of MMP‐9.^32^, ^33^ On the other hand, there is evidence that MMP‐9 may degrade the β‐DG to set free a 30‐kD product from the 43‐kD full‐length subunit.^34^ Furthermore, the proteolysis of dystroglycan by MMP‐9 results in the allosteric regulation of neurexins and the rest of dystroglycan, which in turn produces specific structural and functional changes in the pre‐synaptic and post‐synaptic cytoplasmic environments.^35^ However, the expression level of MMP‐9 and the regulated role of MMP‐9 in dystroglycan degradation in neurons under the high glucose exposure has not been reported.

Based on the above evidence, we suggested that high glucose toxic may induce the β‐DG degradation and aggravate cognitive impairments, such as learning and memory; miR‐132 and MMP‐9 might be involved in the mechanisms underlying of this phenomenon. In this report, we provided several lines of evidence supporting a notion that miR‐132 down‐regulates high glucose‐induced β‐DG degradation through MMP‐9 down‐regulation in primary neurons; ultimately, the changes in the pre‐synaptic and post‐synaptic cytoplasmic environments affect transmission of information among the synapses and cognition function. It might provide valuable insights for elucidating the molecular mechanism and finding therapeutic strategies for the treatment of diabetes‐induced cognitive dysfunction.

## 2 MATERIALS AND METHODS

### 2.1 Animal model and treatment

Specific pathogen‐free (SPF) SD rats aged 4 weeks were purchased from Shanghai Bikai Laboratory Animal Co. Ltd. Rats were housed in SPF environment and given food and water. A 12‐h/12‐h light/dark cycle was maintained. After one month of adaptive feeding, the rats were randomly divided into two groups: (a) sham operated control rats (control group); (b) streptozocin (STZ) and high‐fat diet (HFD) induced type 2 diabetes rats (DM group). The DM group rats were fed with HFD for 4 weeks and then injected once with STZ (in the tail vein at 50 mg/kg bodyweight) to induce partial insulin deficiency, followed by continued HFD feeding for an additional 4 weeks. Then, the rat presence of hyperglycaemia (fasting blood glucose level ≥11.1 mM and non‐fasting glucose levels of ≥16.7 mM) were used for the next experiments. The control group rats were fed with control diet with 10 kcal% fat and injected same dose of saline. All procedures were conducted in accordance with the National Institutes of health guidelines for the care and use of laboratory animals. Make every effort to reduce the number of animals used and their suffering.

Streptozotocin (Cat. No. S0130) was purchased from Sigma Aldrich. High‐fat diet (Cat.D12492) with 60 kcal% fat and control diet (Cat.D12450B) with 10 kcal% fat were purchased from Research Diets.

### 2.2 Morris Water Maze

Spatial learning and memory were assessed by the MWM test. The test consisted of 5‐day training and a probe trial on day 6. Rats were individually trained in a circular pool (diameter 120 cm, height 50 cm) containing 30‐cm high water. The water was rendered opaque by the addition of black non‐toxic ink and maintained at about 25℃. A submerged platform (9 cm in diameter, 2 cm below the surface of water) was placed in the centre of one quadrant of the pool, and its position was fixed throughout the training sessions. There were 5 rats in the control group and the same numbers in DM group. Rats were given four trials of 90 s per day for five consecutive days. Animals were guided to the platform if it was not located within 90 s. All the rats remained on the platform for 10 s before removal. The time (escape latency) that elapsed until the rat reaches the platform was noted each day. After the acquisition phase, rats were given one probe trial of 60 s for which the platform was removed from the pool on day 6. The number of times that the animals swim over the platform location and the pathways that elapsed were evaluated. Data of the escape latency, the number of platform location crossings and the pathways were collected by the video tracking equipment and processed by a computer equipped with an analysis‐management system (‘Dr Rat’ Animal Behavior Analysis System, version 2.20).

### 2.3 Primary neuronal isolation, culture and reagents

**FIGURE 1:**
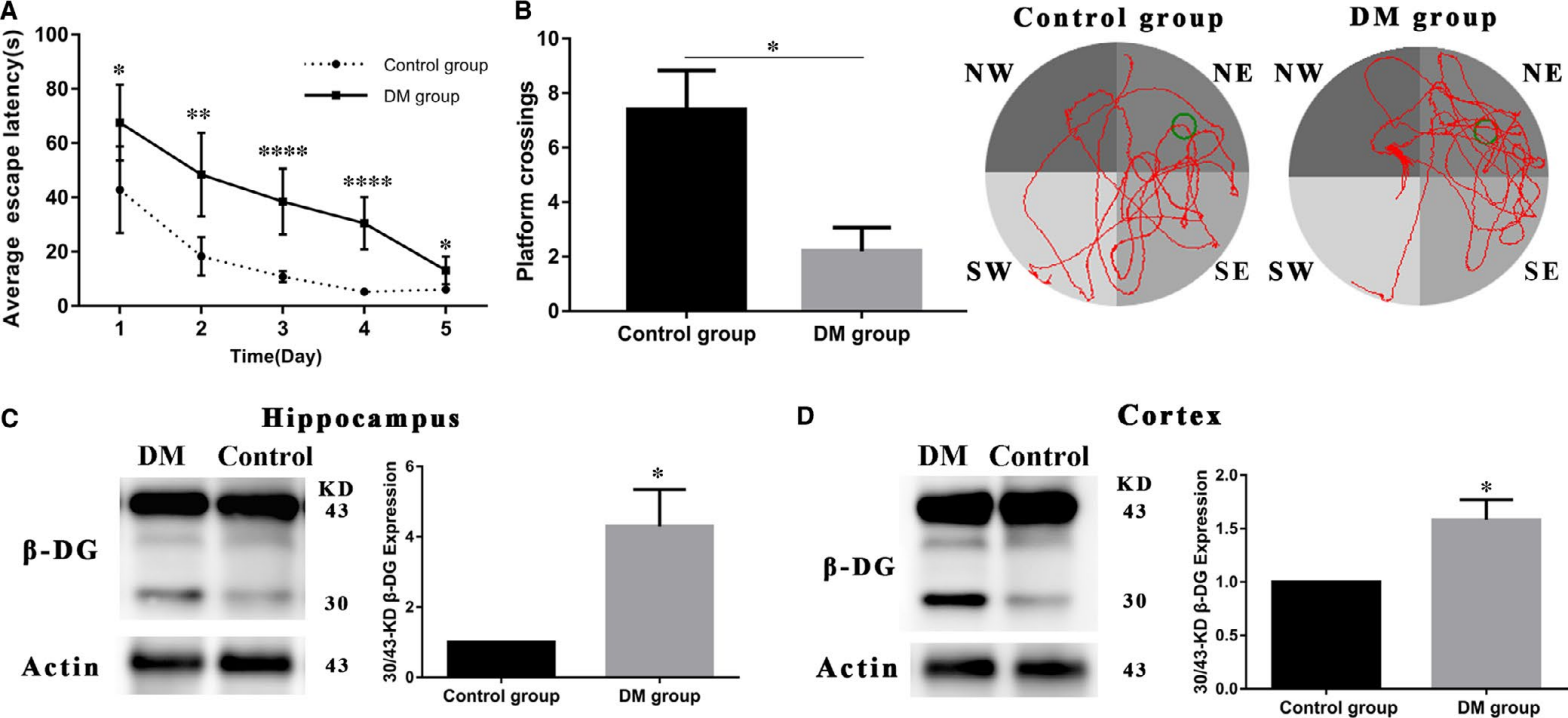
The cognitive abilities and β‐DG in the diabetic rats were detected. (A) Changes in average escape latency to reach the platform during acquisition trials (days 1‐5). (B) The number of times and pathways the rats swim over the hidden platform location (the green circle) during the probe test. (C, D) Hippocampal (C) and cortical (D) tissues were obtained from the diabetic and control rats. Full forms of 43‐kD and degradation forms of 30‐kD β‐DG were detected by Western blot. β‐actin was used as a loading control. Figures showed ratio of 30/43‐kD β‐DG. Data represent mean ± SEM, *P < .05, **P < .01, ***P < .001, ***P < .0001

SPF E18 SD rats aged 10‐12 weeks were purchased from Shanghai Bikai Laboratory Animal Co. Ltd. All procedures were conducted in accordance with the National Institutes of health guidelines for the care and use of laboratory animals. Make every effort to reduce the number of animals used and their suffering. During the experiment, ensure that the pregnant rats are healthy and no stillborn, otherwise they are excluded. The pregnant rats were anaesthetized by carbon dioxide. The methods referred to several articles about inhalation anaesthetics.^36^, ^37^, ^38^ When the rat loses response to toe pinch, the cervical dislocation was carried out. Spray the dam's abdomen area with 75% ethanol. Then, make a incision along the midline of abdomen to expose the uterus. The uterus is then exposed along the midline of the abdomen. Remove the embryos and place individual ones in a petri dish with cold Hank's Balanced Salt Solution (Beyotime). Then, the embryonic brain is dissected and immediately placed in an ice vessel containing Dulbecco's modified Eagle's media (DMEM, Gibco). Hippocampi or cortices tissues were isolated from embryos by using a dissecting microscope. Tissues are digested with 0.125% trypsin for 30 minutes at 37℃, 5% carbon dioxide and shaken every 5 minutes; then, the digested tissues were terminated in DMEM containing 10% FBS (Gibco, BRL, USA). The cell suspension was then filtered through a 40 μm cell strainer and centrifuged at 1000 r/minutes for 5 minutes. The supernatant was removed and resuspend the cells with DMEM containing FBS. The cells were seeded into 6‐well plates or other culture containers coated with 0.1 mg/mL poly‐D‐lysine (Sangon Biotech) at a density of 6‐7 × 10^5^ cells/mL and incubate at 37℃, 5% carbon dioxide for 4‐6 hours. Then, the culture medium was full changed to Neurobasal Medium (Gibco) supplemented with 1× B27 (Gibco) and 1× GlutaMAX (Gibco). Half medium was changed twice a week. The primary hippocampal and cortical neurons on day in vitro (DIV) 1, 3, 5 and 7 were pictured (Figure 1). After 10 days in culture, half of the medium was replaced by fresh medium, and cells were incubated with 25 mM of D‐glucose (yielding a total 50 mM glucose). Then, the cells were maintained for further 48 hours. The corresponding control group was treated with mannitol.

The study protocol was approved by the Animal Care and Use Committee of The Tenth People's Hospital of Shanghai (ID Number: SHDSYY‐2019‐3893) and Tongji University.

### 2.4 Transfection of primary neurons

The miR‐132 mimics and its control, miR‐132 inhibitors and its control, small interfering RNAs (siRNAs) targeting MMP‐9 and the negative control siRNA were designed and constructed by RiboBio. After 7 days culture in vitro, the primary neurons were transfected by using Transfection regent (C10511‐05 riboFECT CP Transfection Kit, RiboBio) according to the manufacturer's instructions. We used MMP‐9 siRNA and miR‐132 mimics at 50 nM and miR‐132 inhibitors at 100 nM final concentration. The MMP‐9 siRNA sequences were CCGGGAACGTATCTGGAAA. The expression of target genes after transfection of siRNA is about 70% according to our experience.

### 2.5 Extraction of total RNA and microRNA

The neurons were gathered, and all RNA were extracted with TRIzol reagent (Invitrogen, Thermo Fisher Scientific) according to the manufacturer's protocol. For all RNA samples, the Nanodrop (Thermo Fisher Scientific) was used to detect the purity and quality.

### 2.6 Reverse transcription and quantitative real‐time polymerase chain reaction (qRT‐PCR) analysis

Reverse transcription of mRNA and microRNA were performed with different primers. For mRNA analysis, cDNA was synthesized using a PrimeScript™ RT reagent kit (Takara RR037A). The real‐time PCR experiment was performed with the real‐time PCR kit (Takara RR820A), which was followed by detection using a 7900HT fast RT‐PCR instrument (Thermo Fisher Scientific) according to the manufacturer's protocol. For the quantitative analysis of miR‐132 expression, Bulge‐Loop miRNA RT Primer was used rather than Oligo dT primer and Random 6 mers in the PrimeScript™ RT reagent kit. Bulge‐Loop miRNA qRT‐PCR Primer was purchased by RiboBio (Guangzhou, China). The relative expression levels of the mRNA and miR‐132 were normalized with GAPDH (Sangon, shanghai, China) or U6, respectively. Expression levels were calculated using the 2^−∆∆^ CT method. The qRT‐PCR primer sequences for MMP‐9 were 5′‐GTCCCTGCTCTACAATGTCC‐3′ (forward) and 5′‐CTTCACTTCCGAGATCTCTTCC−3′ (reverse); the sequences for GAPDH were 5′‐ACCACAGTCCATGCCATCAC‐3′ (forward) and 5′‐TCCACCACCCTGTTGC TGTA−3′ (reverse).

### 2.7 Use of the online tool

The target genes of miR‐132 were predicted using biological databases: PicTar (https://pictar.mdc‐berlin.de/), GSEC (https://www.gsea‐msigdb.org/gsea/index.jsp), microRNA (http://www.microrna.org/microrna/microrna/home.do) and miRSponge (http://bio‐bigdata.hrbmu.edu.cn/miRSponge/). The Venn diagram was drawn by Venny 2.1 (http://bioinfogp.cnb.csic.es/tools/venny/index.html).

### 2.8 ELISA

After the treatment, the supernatant of the culture medium was collected. The concentrations of MMP‐9 were determined using Rat MMP‐9 ELISA Kits (YCBIO). Then, the following procedures were performed following the manufacturer's instructions.

### 2.9 Western Blot

Total proteins of primary neurons were extracted with lysis buffer (100 mM Tris‐HCL, PH 6.8, 4% SDS, 20% glycerol). Proteins (5‐30 μg per lane) were electrophoretically separated on a 10% sodium dodecyl sulphate‐polyacrylamide gel (SDS‐PAGE, Bio‐Rad) and transferred electrophoretically onto PVDF membranes. PVDF membranes were blocked in 10% non‐fat milk for 1 h and then were incubated with anti‐β‐DG (1:200; Santa Cruz Biotechnology), MMP‐9 (1:1000, Wanleibio) and mouse anti‐β‐actin (1:1000; Cell Signaling Technology) overnight at 4℃. Membranes were washed with Phosphate‐buffered saline (PBS) containing 0.1% Tween 20 (PBST) three times and incubated with the secondary antibodies (anti‐mouse IgG, Li Cor Biosciences) for 1 h at room temperature. The membranes were then detected by the Odyssey two‐colour infrared laser imaging system (LI‐COR, Lincoln), or the membranes were processed with enhanced chemiluminescence (ECL) Western blot detection reagents (Millipore, Billerica) and then detected by Amersham Imager 600 (GE).

### 2.10 Gelatin Zymography

The neurons were treated, and the supernatant of the culture medium was collected. The activity of MMP‐9 was determined using MMP gelatin zymography electrophoretic analysis technique. MMP gelatin zymography kit was purchased from GENMED SCIENTIFICS INC. Samples of the culture medium were mixed with APMA (GENMED SCIENTIFICS INC.) and incubated at 37 ℃ for an hour before electrophoresis. Then, the following procedures were performed following the manufacturer's instructions.

### 2.11 Statistical analysis

All experimental procedures strictly follow the principle of randomization and double‐blind. All experiments have been replicated at least 3 times. The Western blot images were quantified with ImageJ Software (version 1.51k, NIH). All of the data were presented as mean ± standard error mean (SEM). Student's t test was performed as appropriate using GraphPad Prism 6. A probability value (*P*) of less than .05 was considered to be statistically significant. All figures were put together in Adobe Photoshop CS6.

## 3 RESULTS

### 3.1 Alterations of cognitive abilities and β‐DG in the diabetic rats

Spatial learning and memory were evaluated on the basis of MWM performance in diabetes rats (DM group) and sham operated control rats (control group). The behavioural training protocol is presented in MWM method. The time (escape latency) that elapsed until the rat reaches the platform was noted during acquisition trials (days 1‐5). The number of times that the animals swim over the platform location and the pathways that elapsed were evaluated during the probe test (day 6). We compared learning curves of two group rats. As shown in Figure 1A, the average escape latency of the diabetes rats was longer than those of control rats every day during the learning phase. A considerable difference was also observed between the performance of diabetes and control rats on day 3 and 4. As depicted in Figure 1B, during the probe test, the diabetes rats crossed the platform less often than the control rats; control rats spent significantly more time in the platform quadrant (the green circle) whereas diabetes animals swam randomly. We suggested that the spatial learning and memory decline in diabetic rats was related to dystroglycan protein degradation. Thus, we took out hippocampal and cortical tissues from the two group rats to detect the changes of β‐DG protein. The full‐length forms of 43‐kD and degradation forms of 30‐kD β‐DG were detected by Western blot. Higher levels of 30/43‐kD β‐DG (*P* < .05) were observed in the diabetic rats compared to the control group (Figure 1C,D). Thus, we concluded that the toxic effects of hyperglycaemia could significantly induce β‐DG degradation of in both hippocampal and cortical tissues in brain.

### 3.2 High glucose induces β‐DG degradation in primary neurons

**FIGURE 2:**
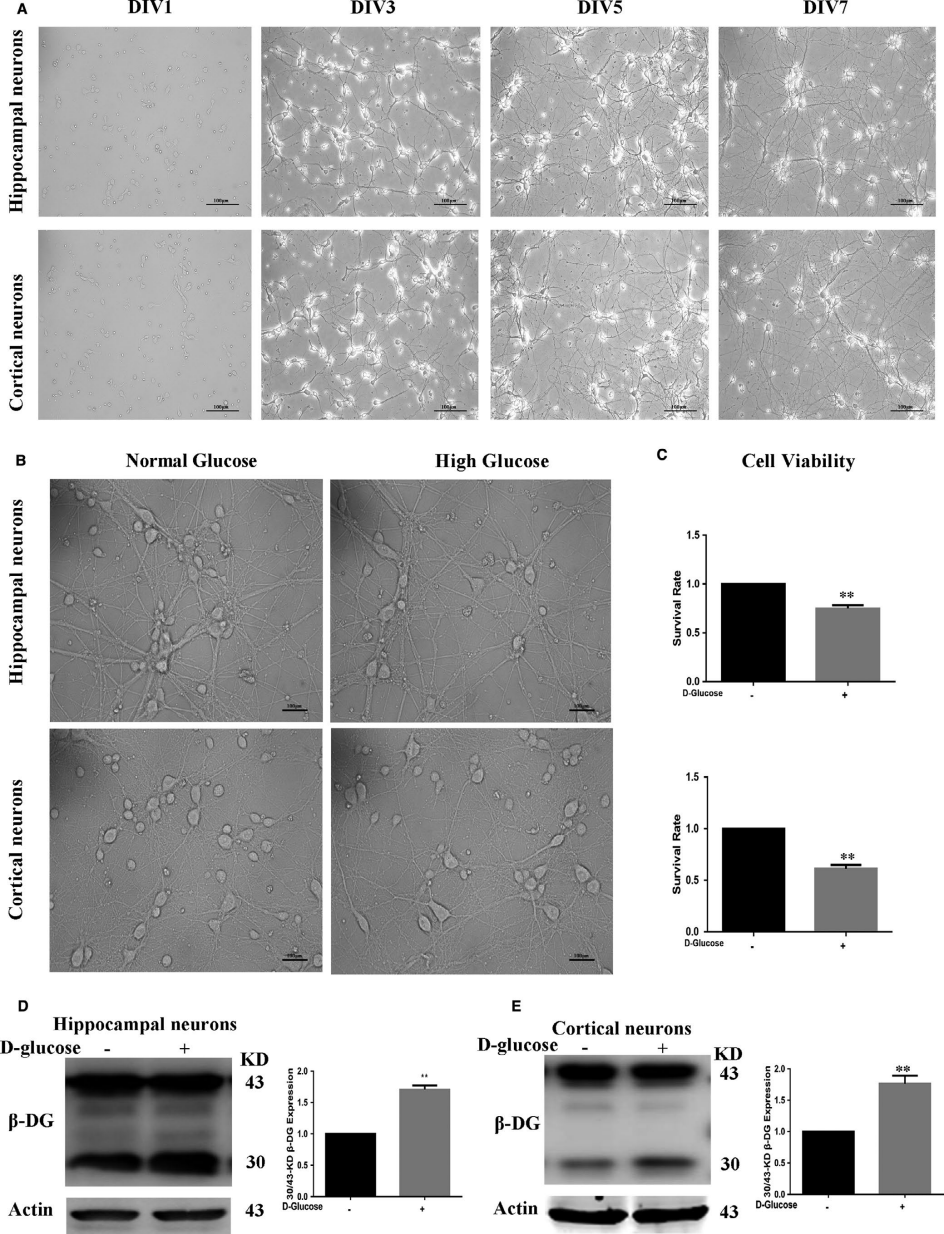
The primary neurons were cultured in vitro, and the effects of high glucose exposure on them were detected. (A) Images of cultured primary neurons. Images of cultured hippocampal and cortical neurons at different stages during culture—DIV 1, 3, 5 and 7. Note how neuronal morphology and arborization develop over time. Scale bar = 100 μm (B, C) Primary neurons were treated with high glucose. The control group was treated with mannitol to adjust osmotic pressure. No obvious changes in neuron morphology observed through a microscope. Scale bar = 100 μm (B). Cell viability of neurons with high glucose treatment was lower than that of the control group (C). (D, E) Western blot was used to detect the full‐length 43‐kD β‐DG and the degradation 30‐kD β‐DG. Figures showed higher levels of 30/43‐kD β‐DG in the high glucose group compared to the control normal glucose group. Data represent mean ± SEM. *P < .05, **P < .01

To investigate the ‘toxic’ effects of hyperglycaemia on neurons, we cultured hippocampal and cortical neurons and the cultures were treated with high glucose for 48 h. The control group was treated with mannitol to adjust osmotic pressure. As shown in Figure 2B, there were no obvious changes in neuron morphology observed through a microscope. Cell viability of primary neurons with high glucose treatment was lower than that of the control group (Figure 2C). Based on the above hypothesis, we verified the high glucose effect on β‐DG degradation. Western blot was used to detect the full‐ length 43‐kD β‐DG and the degradation 30‐kD β‐DG, in high glucose and normal glucose groups. In Figure 2D,E, higher levels of 30/43‐kD β‐DG were observed in the high glucose group compared to the control normal glucose group. As a result, we concluded that high glucose could significantly induce the degradation of β‐DG in both primary hippocampal and cortical neurons.

### 3.3 High glucose decreases miR‐132 expression in primary neurons and miR‐132 down‐regulates high glucose‐induced β‐DG degradation in primary neurons

**FIGURE 3:**
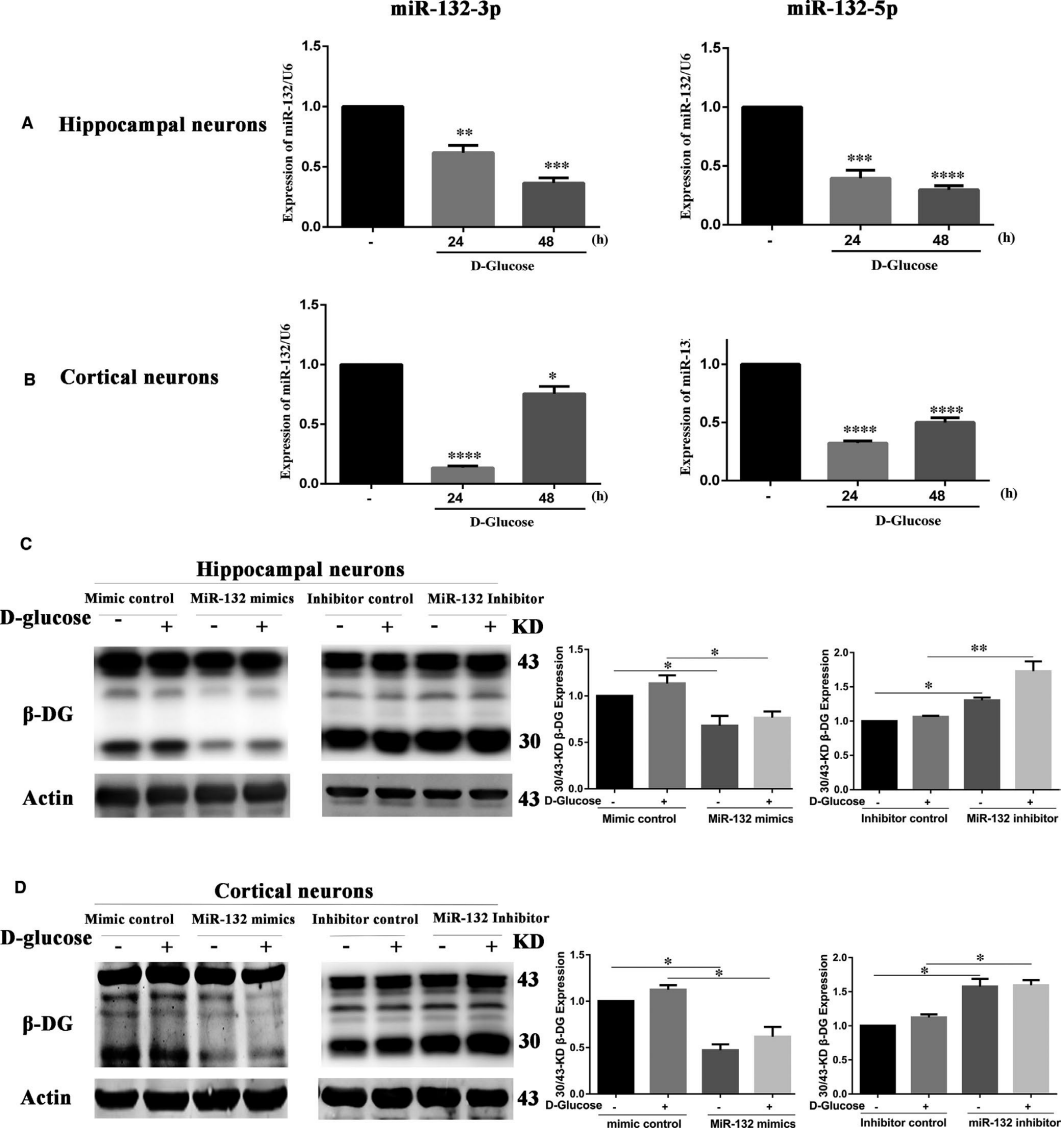
High glucose decreased miR‐132 expression, and miR‐132 down‐regulated high glucose‐induced β‐DG degradation. (A, B) Primary neurons were treated with high glucose for 24 or 48 h. The miR‐132‐3p and 5p expression in hippocampal (A) and cortical neurons (B) was analysed. (C, D) Primary neurons were transfected with a mimic negative control, miR‐132 mimic, an inhibitor negative control, or miR‐132 inhibitor, followed by stimulation high glucose. β‐actin was used as a loading control. The 30 and 43‐kD β‐DG levels in hippocampal (C) and cortical neurons (D) were detected by Western blot. Figures showed the ratio of 30/43‐kD β‐DG. Data represent mean ± SEM. *P < .05, **P < .01, ***P < .001, ****P < .0001

Pre‐miRNA has two arms, 5p and 3p, respectively. In many cases, only one arm is processed as a mature miRNA. However, in some cases, both 5p and 3p can be expressed as mature miRNA. The 5p and 3p have different expression profiles and biological functions. To determine the expression of miR‐132 in primary cortical and hippocampal neurons under the high glucose exposure, the neurons were treated with high glucose for 24 or 48 h. The results showed that both miR‐132‐3p and miR‐132‐5p expressions decreased in primary hippocampal and cortical neurons under the high glucose exposure in Figure 3A,B. The microRNA expression has time and tissue specificity. Interestingly, miR‐132 expressions upon glucose treatment are different between hippocampal and cortical cultures. Only in cortical neurons, the decrease of miR‐132 was much larger after 24 hours than after 48 hours. In hippocampal neurons, miR‐132 decreased with the increase of the high glucose exposure time. As a result, we confirmed that miR‐132 was significantly down‐regulated by high glucose stimulus.

To elucidate the role of miR‐132 in β‐DG degradation, primary neurons were transfected with a mimic negative control, miR‐132 mimic, an inhibitor negative control, or miR‐132 inhibitor, followed by stimulation with high glucose. Western blot was used to detect the β‐DG degradation. As shown in Figure 3C, in hippocampus neurons, transfection with miR‐132 mimics significantly down‐regulated the ratio of 30/43‐kD β‐DG compared to the mimic negative control. In contrast, the miR‐132 inhibitor enhanced the ratio of the 30/43‐kD β‐DG compared to the inhibitor negative control. A similar pattern was observed in cortical neurons in Figure 3D. It revealed that the miR‐132 could down‐regulate the glucose‐induced β‐DG degradation in primary neurons. Above all, these results imply that miR‐132 participates and negatively affects glucose‐induced β‐DG degradation.

### 3.4 High glucose increase the MMP‐9 expression via decreasing the levels of miR‐132

**FIGURE 4:**
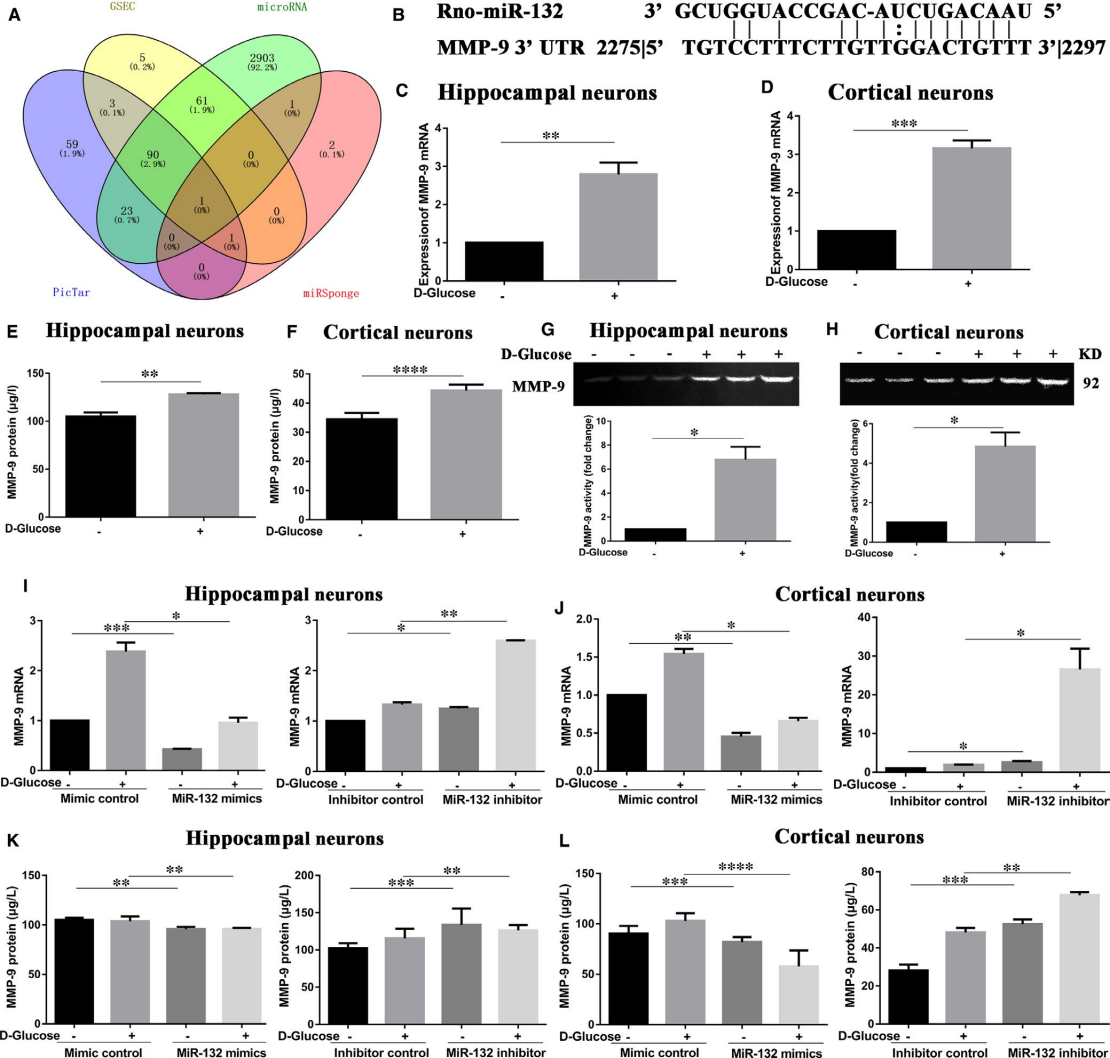
MMP‐9 was confirmed to be the target of miR‐132 in neurons. (A) Useful information of PicTar, GSEC, microRNA and miRSponge was summarized to draw the Venn diagram. (B) The binding sites between miR‐132 and MMP‐9 mRNA in neurons have reported. (C‐F) Primary hippocampal and cortical neurons were treated with high glucose. QRT‐PCR analysis showed MMP‐9 mRNA expression in hippocampal neurons (C) and cortical neurons (D). ELISA analysis showed the MMP‐9 protein expression in hippocampal neurons (E) and cortical neurons (F). Gelatin zymography analysis the MMP‐9 activity in hippocampal neurons (G) and cortical neurons (H). (I‐L) Transfected miR‐132 mimics or miR‐132 inhibitor into neurons and then followed by stimulation with high glucose. Levels of MMP‐9 mRNA were measured by qRT‐PCR in hippocampal neurons (I) and cortical neurons (J). Levels of MMP‐9 protein were measured by ELISA in hippocampal neurons (K) and cortical neurons (L). Data represent mean ± SEM. *P < .05, **P < .01, ***P < .001, ****P < .0001 compared with corresponding control group

As mentioned above, miRNAs mostly promote mRNA degradation and translation by binding to the sequences of target mRNA. To explore the regulatory functions of miR‐132 in the high glucose‐induced β‐DG degradation, we screened authoritative databases of predicting target genes of miRNAs to identify target genes of the miR‐132. Thus, we looked through the following databases: PicTar, GSEC, microRNA and miRSponge. Useful information from four databases was summarized to draw the Venn diagram (Figure 4A). The results showed that miR‐132 may directly bind to the family of MMP. In addition, it has reported that miR‐132 can directly bind to the 3’ untranslated region (UTR) of the MMP‐9 mRNA in neurons. The binding site is shown in Figure 4B.^29^

Thus, we suggested that there were regulatory functions of MMP‐9 in the high glucose‐induced β‐DG degradation. It has been reported that MMP‐9 is produced at the synapse and released into the extracellular space in a precursor form, and then, it is activated by proteolytic cleavage.^34^ It was demonstrated that only secreted and activated MMP‐9 causes the degradation of its extracellular substrates. Therefore, we used ELISA, Western blot and gelatin zymography to detect the expression and activity levels of MMP‐9 protein in cell culture. The results in Figure 4C‐H and Figure S1A,B showed that high glucose could significantly up‐regulated MMP‐9 mRNA and protein expression, as well as protein activity in primary neurons. In detail, the qRT‐PCR analysis showed MMP‐9 mRNA expression in hippocampal (Figure 4C) and cortical neurons (Figure 4D) under high glucose exposure was significantly higher than those of control one. ELISA and Western blot analysis showed the MMP‐9 protein expression in hippocampal (Figure 4E, Figure S1A) and cortical neurons (Figure 4F, Figure S1B) under high glucose treatment was higher than those of control group. Gelatin zymography analysis showed the MMP‐9 activity in hippocampal (Figure 4G) and cortical neurons (Figure 4H) exposed under high glucose was higher than those of control one.

In order to validate that MMP‐9 is miR‐132 target gene in neurons under high glucose exposure, we transfected miR‐132 mimics or miR‐132 inhibitor into hippocampal and cortical neurons and then followed by stimulation with high glucose. The qRT‐PCR (Figure 4I,J), Western blot (Figure S1C,D) and ELISA data (Figure 4K,L) indicated that the miR‐132 mimics down‐regulated MMP‐9 mRNA and protein expression compared to the mimic negative control under the same glucose concentration, whereas the miR‐132 inhibitor up‐regulated MMP‐9. Therefore, data from previous studies and our findings indicated that MMP‐9 mRNA was a target of miR‐132 in primary neurons exposed by high glucose. Through the above experiments, we concluded that high glucose could increase the expression of MMP‐9 via decreasing the levels of miR‐132.

### 3.5 Suppression of MMP‐9 inhibits high glucose‐induced β‐DG degradation in primary neurons

**FIGURE 5:**
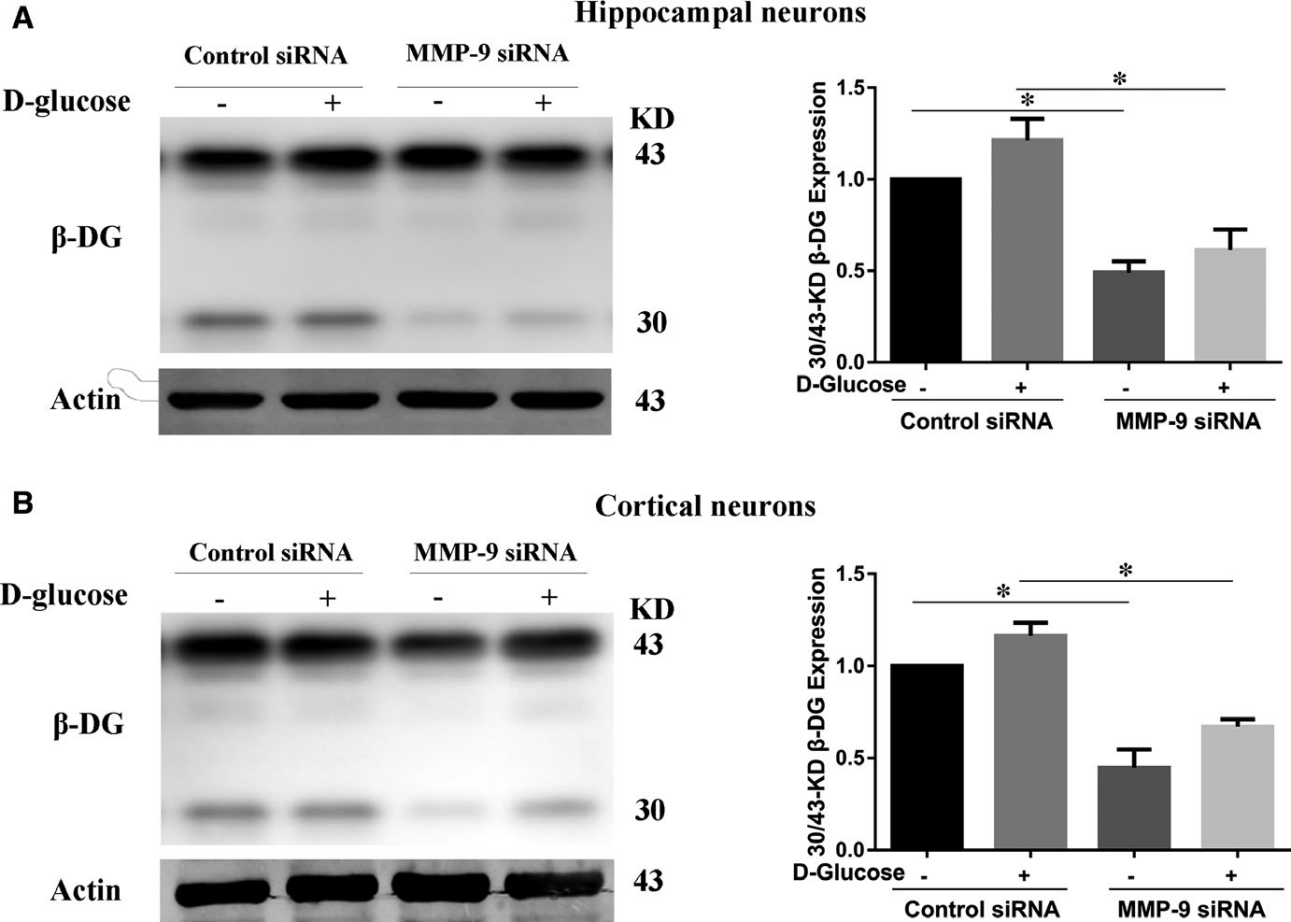
Suppression of MMP‐9 could inhibit high glucose‐induced β‐DG degradation in neurons. (A, B) Primary neurons were transfected with MMP‐9 siRNA or control siRNA and then treated with high glucose. The 30 and 43‐kD β‐DG levels in hippocampal neurons (A) and cortical neurons (B) were examined by Western blot analysis. The graphs showed the ratio of 30/43‐kD β‐DG expression. Data represent mean ± SEM. *P < .05 compared with the control siRNA‐transfected neurons under the same glucose concentration

It has reported that MMP‐9 may digest the β‐DG to release a 30‐kD product from the 43‐kD full‐length subunit and play an important role in synaptic plasticity. To understand the direct influence of MMP‐9 on high glucose‐induced β‐DG degradation, primary hippocampal and cortical neurons were transfected with MMP‐9 siRNA or control siRNA and then treated with high glucose. Then, the 30 and 43‐kD β‐DG were examined by Western blot. The graphs in Figure 5 showed the ratio of 30/43‐kD β‐DG detected by Western blot. MMP‐9 siRNA‐transfected neurons exhibited a significant decrease in 30/43‐kD β‐DG compared to the siRNA control under the same glucose concentration. It meant that the suppression of MMP‐9 could down‐regulate high glucose‐induced β‐DG protein degradation. From these results, we concluded that MMP‐9 could contribute to β‐DG protein degradation under the toxic of high glucose.

### 3.6 The miR‐132 inhibits the degradation of β‐DG via down‐regulation of the expression level of MMP‐9

**FIGURE 6:**
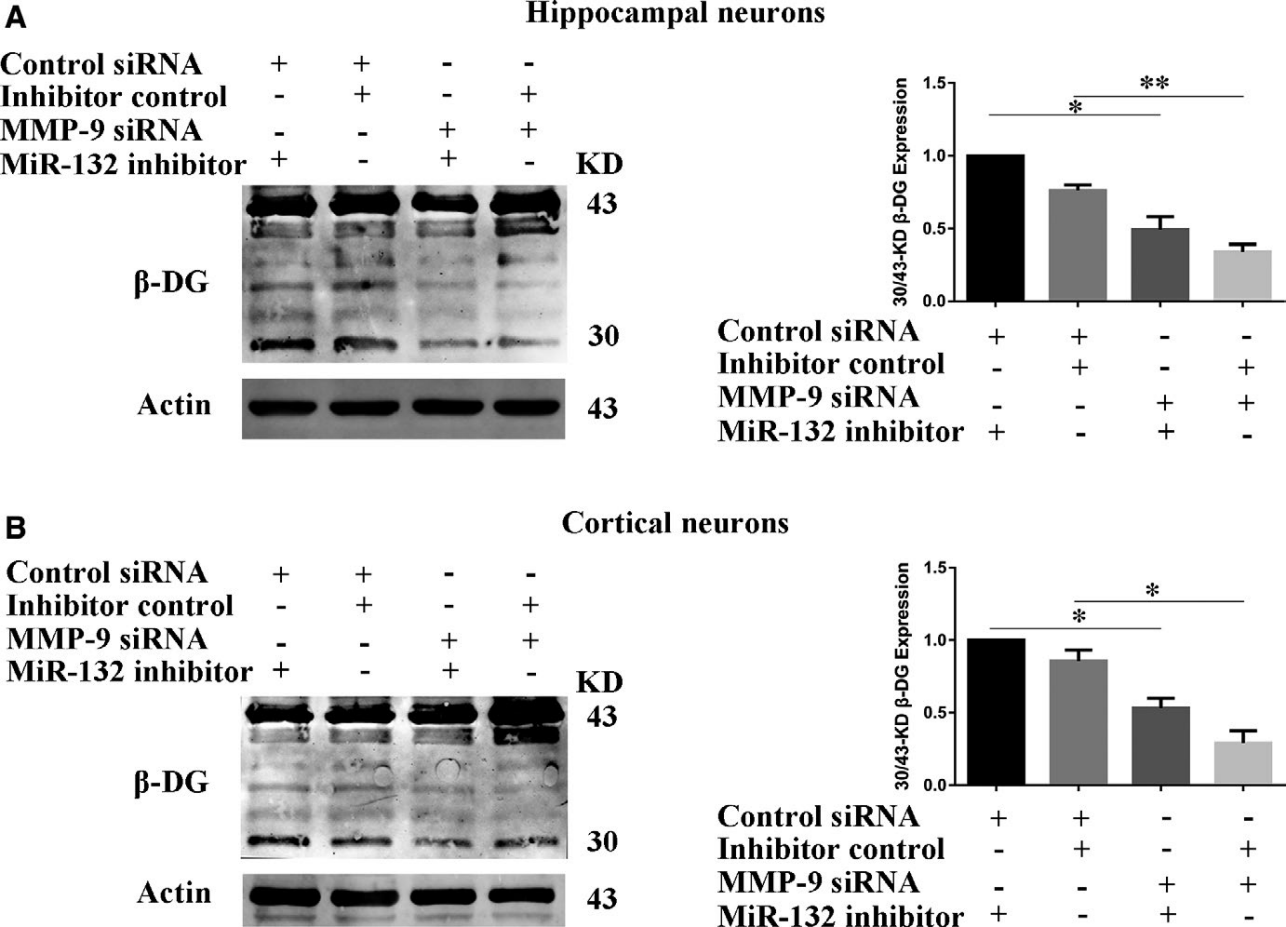
The miR‐132 could inhibit β‐DG degradation via down‐regulation of MMP‐9 expression levels. (A, B) Co‐transfect primary neurons with MMP‐9 siRNA and miR‐132 inhibitor. Then, the β‐DG expression in hippocampal neurons (A) and cortical neurons (B) was examined by Western blot. The graphs showed the ratio of 30/43‐kD β‐DG expression. Data represent mean ± SEM. *P < .05, **P < .01 compared between two groups

Follow on, we examined whether β‐DG degradation through miR‐132 inhibitor was attenuated by the knockdown of MMP‐9. Co‐transfect primary neurons with MMP‐9 siRNA and miR‐132 inhibitor for 48h. Then, the β‐DG expression in neurons was examined by Western blot. The graphs in Figure 6 showed the ratio of 30/43‐kD β‐DG detected by Western blot. As shown in Figure 6A, co‐transfection with MMP‐9 siRNA, but not control siRNA, significantly diminished the increase of 30/43‐kD β‐DG by miR‐132 inhibitor in hippocampus neurons. A similar pattern was observed in cortical neurons (Figure 6B). These findings showed that the knockdown of MMP‐9 could counteract the β‐DG degradation effect by inhibition of miR‐132; it further indicated that miR‐132 could regulate β‐DG degradation through down‐regulation of MMP‐9.

### 3.7 Schematic diagram of the proposed model shows how high glucose‐induced β‐DG degradation

**FIGURE 7:**
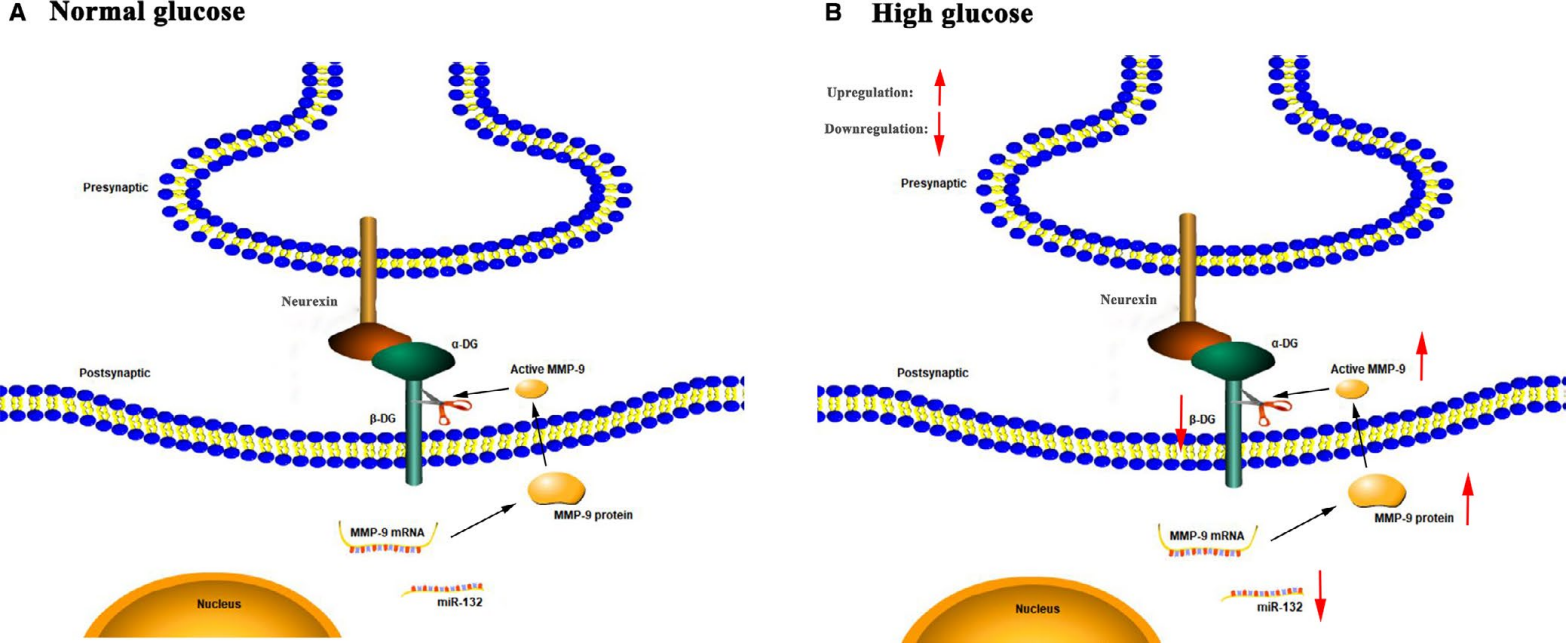
The schematic diagram showed the effect of miR‐132 and MMP‐9 on β‐DG degradation under high glucose exposure. At the steady state (A), dystroglycan protein extends from the post‐synaptic side into the ECM to binds neurexins, which located in the pre‐synaptic sides. When neurons are exposed to high glucose environment in long‐term period (B), miR‐132 would be down‐regulated in neurons. MMP‐9 mRNA, as a target of miR‐132, could be up‐regulated; higher expression and overlay activity of MMP‐9 protein could increase β‐DG protein degradation. In this way, high glucose could induce the β‐DG degradation, consequently might affect synaptic plasticity and neuronal signalling, as well as cognitive impairment, such as learning and memory. Ultimately, β‐DG degradation may affect structure and functions among the synapses, which related to cognition decline

Above all of our findings, we proposed the model of high glucose inducing β‐DG degradation. The schematic diagram was showed in Figure 7. When neurons are exposed to high glucose environment in long‐term period, miR‐132 would be down‐regulated in neurons. MMP‐9 mRNA, as a target of miR‐132, could be up‐regulated; higher expression and overlay activity of MMP‐9 protein could increase β‐DG protein degradation. In this way, β‐DG degradation may affect structure and functions among the synapses, which related to cognition decline.

## 4 DISCUSSION

In a stable state, pre‐synaptic and post‐synaptic connections are made directly by transmembrane or ECM molecules, such as neurexin and dystroglycan protein. When the neurotransmitters are released from the pre‐synaptic to the post‐synaptic sides, active MMPs are released to perform limited proteolysis of dystroglycan.^35^ In this way, MMPs and dystroglycan play an essential role on structure and function changes and signal transmission in the synapsis‐ECM environment. Here, we find the presence of high glucose can induce the β‐DG degradation and report a potential mechanism of miR‐132 and MMP‐9 in the regulation of high glucose‐triggered β‐DG degradation in primary neurons. Our results showed that high glucose induced the enhancement of β‐DG degradation, which consequently might affects synaptic plasticity and neuronal signalling, as well as cognitive function, such as learning and memory. In addition, miR‐132 served as a negative feedback regulator in high glucose‐induced β‐DG degradation via its target MMP‐9 down‐regulation.

DM is associated with cognitive dysfunction, which seriously affects the quality of life of patients. Its pathophysiological mechanisms are complex and diverse. One of them of is that hyperglycaemia toxic could damage the synaptic proteins and impede the transmission of information between synapses. Unfortunately, there are currently no effective methods to block its progression.^39^ In this study, to investigate the ‘toxic’ effects of hyperglycaemia, we made diabetic rat model and treated the primary hippocampal and cortical neurons with high glucose. Our results showed that spatial learning and memory of diabetes rats significantly declined compared to that of control group; high glucose significantly induced the degradation of β‐DG, compared to the normal glucose group, in both hippocampal and cortical neurons. Emerging evidence suggests that deficient in dystroglycan protein is involved in cognitive impairment, such as AD. It has also proved that dystroglycan might have a post‐synaptic role in cognitive impairment, such as learning and memory. Moreover, deficient in dystroglycan protein has insufficient long‐term potentiation effect and synaptic plasticity in the brain.^14^, ^15^ Subsequently, neuronal activity decreases and cognitive impairment occurs.

MicroRNA is essential for the normal development of brain and its function.^40^ The miR‐132 in neurons has been widely reported to play an important role in the development of nervous system.^41^ In our report, high glucose down‐regulated the expression of miR‐132. This is consistent with previous studies that miR‐132 is down‐regulated in AD patient brain.^25^ Besides, we found an interesting phenomenon: miR‐132 expressions upon glucose treatment are different between hippocampal and cortical cultures. Only in cortical neurons, the decrease of miR‐132 was much larger after 24 hours than after 48 hours. In hippocampal neurons, miR‐132 decreased with the increase of the high glucose exposure time. However, compared with the control group, the miR‐132 content both decreased after 24 and 48 hours of high glucose treatment. The different effect of high glucose on miR‐132 in cortical and hippocampal neurons has not been reported. We guess the reason for this phenomenon is the difference of steady‐state regulation ability. Cell homeostasis is a kind of dynamic balance. When we treated neurons with high glucose, cell homeostasis would be destroyed and the cells would initiate protection through a series of complex mechanisms. In cortical neurons, the miR‐132 content under 48 hours of high glucose treatment is higher than that under 24 hours. But the miR‐132 content is opposite in hippocampal neurons. We speculate that cortical neurons have a stronger ability to regulate homeostatic imbalance compared hippocampal neurons under 48 hours of treatment. In this way, miR‐132 content could be compensatory increased, but it was still lower than that of the control group.

The miR‐132 can also affect the expression of some synapse‐associated proteins. In this study, we found that the miR‐132 decreased the glucose‐induced β‐DG degradation. These experiment results imply that miR‐132 is involved in glucose‐induced β‐DG degradation and serves as a negative feedback regulator. Previous studies also demonstrated that overexpression of miR‐132 could protect the neurons. This protection improves learning and memory.^42^

It has been known that MMP‐9 is involved in synaptic plasticity in cognitive processes. The abnormal MMP‐9 activity may lead to cognitive dysfunction.^32^ Our study found that high glucose significantly increased the expression and activity levels of MMP‐9 in primary neurons. It is not inconsistent with those previous reports that up‐regulation of MMP‐9 can aggravate many neurological diseases, including stroke, PD and AD.^30^, ^43^ Moreover, previous studies and our findings indicated that MMP‐9 mRNA was the target of miR‐132 in neurons. Thus, we further verified that high glucose increased the expression of MMP‐9 via inhibiting the levels of miR‐132. Moreover, our data indicated that miR‐132 regulated β‐DG degradation through down‐regulation of MMP‐9. Other studies also had shown that the specific metalloproteinase activity of β‐DG was associated with MMP‐9.

Through the above experiments, our results showed that high glucose significantly increased the degradation of β‐DG in primary neurons. We further explored the mechanisms underlying of this phenomenon. In summary, we concluded that miR‐132 could down‐regulate high glucose‐induced β‐DG degradation through MMP‐9 down‐regulation in neurons; ultimately, β‐DG degradation might affect structure and functions among the synapses, which related to cognition decline. It may provide some theoretical basis for elucidating the molecular mechanism of diabetes‐induced cognitive dysfunction. At the same time, it also provides new ideas for clinical treatment of diabetes‐induced cognitive dysfunction.

Although we believe the experiment is adequate and the argument is sufficient, there are still some shortcomings and disputes. Firstly, in the process of culturing neurons, the primary neurons gradually mature after 5 days of culture.^44^, ^45^, ^46^ We found that the neurons became stable and mature around day 7. In most of the literatures with similar purposes, the authors chose to treat neurons cultured for about 7 days.^47^, ^48^ Thus, we chose to intervene at this time. Although we believed that the cells were intervened during the optimal processing period, there were indeed limitations. Our results were validated on the 10‐day‐old neuronal cultures, but not on more mature or immature neurons. In addition, the dystroglycan protein is composed of two non‐covalently associated subunits: α‐DG and β‐DG.^8^ However, we only explored the β‐DG degradation under the high glucose but ignored the changes of α‐DG. Current research suggests that the functions of two subunits are consistent.^10^, ^12^ Thus, it can be assumed that if β‐DG subunit is damaged, the structure and function of the dystroglycan protein could be damaged. Yet it is warranted to examine the new theories about dystroglycan in the inter‐neuronal junction and to subsequently check for possible α‐DG changes under high glucose toxic in the future. Besides, a small increase in cell death was detected in neurons exposed to high glucose in our experiment, which is consistent with many previous studies where apoptosis was found in neural cells exposed to high glucose ^47^, ^48^, ^49^, ^50^, ^51^ and in the hippocampus of diabetic animals.^52^, ^53^ But it has been reported in the literature that 50mmol/L HG Neurobasal Medium does not cause cell death.^54^ Thus, the methods of high glucose treatment in each literature were not exactly the same, such as the high glucose treatment time and the purity of neurons, and the results might be different. As long as it is handled properly, the results are reliable.

## CONFLICT OF INTEREST

The authors declare that they have no competing interests.

## AUTHOR CONTRIBUTIONS

**Yunxiao Dou:** Validation (equal); Writing‐original draft (lead). **Yan Tan:** Resources (equal); Writing‐review & editing (equal). **Yanxin Zhao:** Project administration (equal). **xueyuan Liu:** Funding acquisition (equal); Project administration (lead). **Tongya Yu:** Supervision (equal). **Xiaoye Ma:** Supervision (equal). **Yuchen Zhou:** Supervision (equal). **Yichen Zhao:** Supervision (equal).

## Supporting information

Figure S1

## Data Availability

The data that support the findings of this study are available from the first author upon reasonable request.
