# Supplementary figures and images for: MiR‐132 down‐regulates high glucose‐induced β‐dystroglycan degradation through Matrix Metalloproteinases‐9 up‐regulation in primary neurons
Source: J Cell Mol Med. 2021 Jun 23;25(16):7783–95. doi: 10.1111/jcmm.16669 (PMC8358889; doi:10.1111/jcmm.16669)

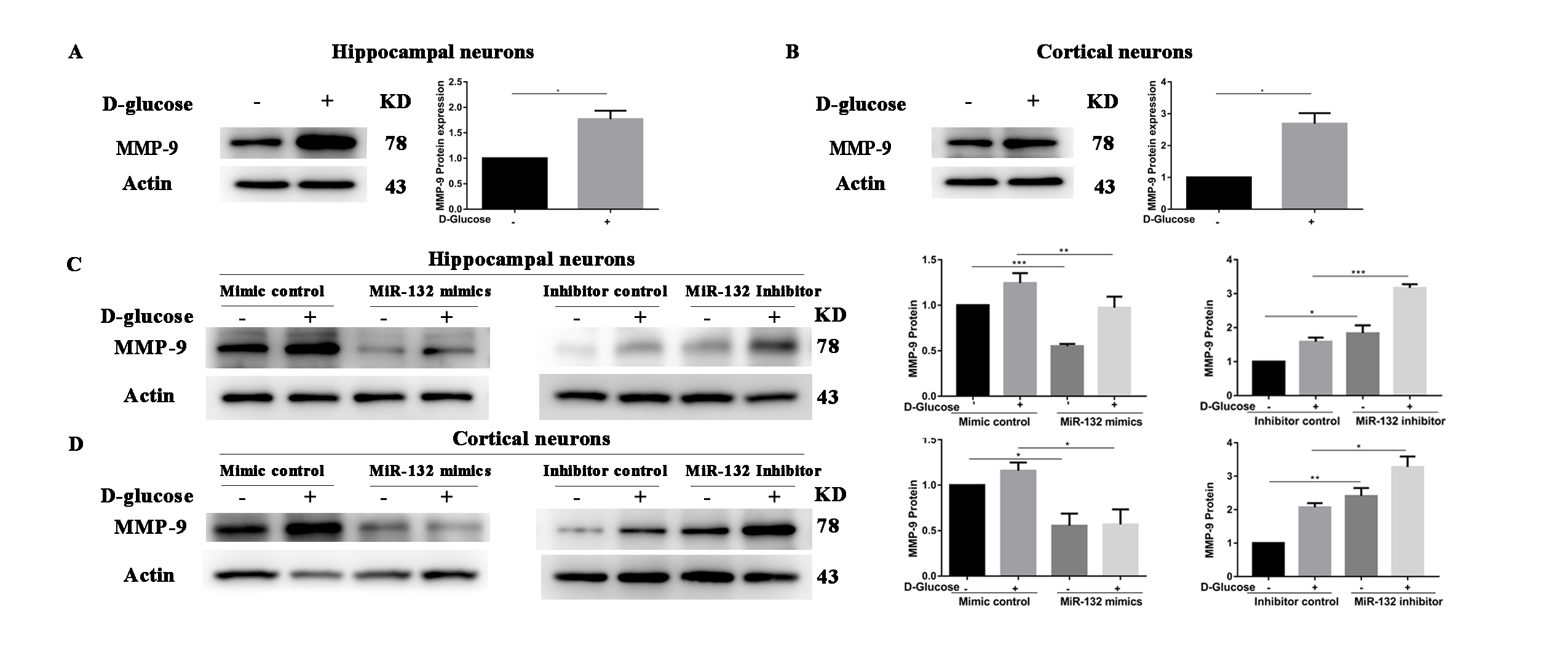

Supplement: Supplementary file 1 — Figure S1 [file JCMM-25-7783-s001.tif]
